# *Yokenella regensburgei*—Past, Present and Future

**DOI:** 10.3390/antibiotics13070589

**Published:** 2024-06-26

**Authors:** Dan Alexandru Toc, Carmen Costache, Vlad Sever Neculicioiu, Irina-Maria Rusu, Bogdan-Valentin Roznovan, Alexandru Botan, Adelina Georgiana Toc, Pavel Șchiopu, Paul-Stefan Panaitescu, Adrian Gabriel Pană, Ioana Alina Colosi

**Affiliations:** 1 Department of Microbiology, Iuliu Hatieganu University of Medicine and Pharmacy, 400012 Cluj-Napoca, Romania; 2 Faculty of Medicine, Iuliu Hatieganu University of Medicine and Pharmacy, 400012 Cluj-Napoca, Romania

**Keywords:** Gram-negative bacteria, *Enterobacteriaceae*, bacterial infections, human infections, veterinary infections, antimicrobial drug resistance, Antimicrobial Susceptibility Testing, virulence factors, ecology, microbiome

## Abstract

*Yokenella regensburgei* is a Gram-negative rod part of the *Enterobacteriaceae* family (order *Enterobacterales*) and a rare cause of human infections. Although improved diagnostic methods have led to an increase in reports of this elusive pathogen, information remains limited. In order to provide a better understanding of this bacterium, we developed the first comprehensive review of its biology, biochemical profile, antimicrobial resistance pattern, virulence factors, natural reservoir and involvement in various veterinary and human infections. Human infections with this bacterium are scarcely reported, most probably due to constraints regarding its identification and biochemical similarities to *Hafnia alvei*. Multiple systematic searches revealed 23 cases of human infection, with a seemingly worldwide distribution, mostly in middle-aged or elderly male patients, often associated with immunosuppression. To date, *Y. regensburgei* has been reported in skin and soft tissue infections, bacteremia and sepsis, osteoarticular infections and in others such as urinary tract and digestive infections. The unique ability of *Y. regensburgei* to degrade polystyrene presents a novel and promising avenue for addressing plastic pollution in the near future. However, large-scale applications of this bacterium will undoubtedly increase human exposure, highlighting the necessity for comprehensive research into its role in human and veterinary infections, pathogenicity and antibiotic resistance.

## 1. *Yokenella regensburgei*—A Brief History

*Y. regensburgei* is a Gram-negative rod, part of the *Enterobacteriaceae* family (order *Enterobacterales*). It was recognized as a distinct species in 1984 when a Japanese team of researchers led by Kosako et al. investigated a collection of bacteria with unusual characteristics from the National Institute of Health (NIH), grouped in NIH biogroup 9 [1].

In their initial study, Kosako et al. analyzed a collection of bacterial strains isolated from insects in order to confirm their initial diagnosis of *Hafnia alvei* [1]. They concluded that 6 out of the initial 30 strains analyzed were not *Hafnia alvei*. Based on their biochemical profiles, these six strains were classified into NIH biogroup 9. Furthermore, all eleven strains included in the NIH collection (six isolates from insects and five isolates from human samples) underwent extensive biochemical characterization. Phenotypically, all eleven strains included in NIH biogroup 9 at that time were Gram-negative, non-spore-forming, non-pigmented, fermentative bacilli with a positive catalase reaction and a negative oxidase reaction. Nearly all strains were motile, and electron microscopy revealed that they possessed peritrichous flagella [1]. Additionally, the use of several supplementary biochemical tests was recommended to better characterize these strains. The use of citrate, a negative Voges–Proskauer reaction and the production of several enzymes like decarboxylases (lysine and ornithine) and beta-galactosidase could differentiate between the NIH biogroup 9 and *Hafnia alvei* and *Salmonella arizonae* [1]. This differential diagnosis was particularly important at that time because the commercially available diagnostic tools were not able to distinguish between these pathogens. Their findings concluded with the emergence of a new genus and species named *Y. regensburgei* [1].

There has been some controversy in the literature regarding the nomenclature of this new and emerging pathogen. In 1985, based on another study conducted by Farmer et al., the Centers for Disease Control and Prevention (CDC) proposed the name *Koserella trabulsii* to the Enteric group 45 [2]. However, it was proved in 1987 by Kosako et al. that *Y. regensburgei* and *Koserella trabulsii* were in fact the same bacteria. In 1991, the CDC admitted that the name *Yokenella regensburgei* had priority due to its earlier publication, leading to the abandonment of the name *Koserella trabulsii* [3,4,5,6].

The name of the newly discovered bacteria has an interesting origin. Kosako et al. explained that the genus name was derived from the term “Yoken”, the Japanese abbreviation for the NIH Tokyo, combined with the feminine diminutive “ella”. The species name, “regensburgei”, reflects the location from which the strains were initially isolated from insects, namely Regensburg, Germany [1].

## 2. Antimicrobial Resistance

The antimicrobial resistance profile of *Y. regensburgei* is in continuous development since precise data are currently lacking. Currently, there are no expert rules or expected resistant phenotypes reported by the European Committee on Antimicrobial Susceptibility Testing—EUCAST (www.eucast.org, accessed on 27 May 2024). Given the fact that *Y. regensburgei* is a member of the *Enterobacterales* group, antibiotic susceptibility testing can be performed and interpreted according to the standard guidance for this group of bacteria.

Only a limited number of available studies have comprehensively analyzed the antimicrobial resistance patterns of this bacterium. In the first reported case series of human infection with *Y. regensburgei*, Abott et al. analyzed the antimicrobial sensitivity of a single strain [7]. Similarly, Hickman-Brenner et al. analyzed the resistance patterns of only 12 strains of *Y. regensburgei* to 12 antibiotic agents, revealing resistance to several antibiotics such as penicillin G, ampicillin and colistin through the disk diffusion technique [6]. Based on these data, the initial hypothesis was formed that these strains may produce a beta-lactamase enzyme [6]. The first extensive antimicrobial resistance profiles of multiple *Y. regensburgei* isolates were first detailed in 2003 by Stock et al. through the broth microdilution method [8]. Based on these results, they were able to present a list of antibiotics for which the bacterial strains are sensitive, like chloramphenicol, fosfomycin, nitrofurantoin, tetracycline, all aminoglycosides and the tested quinolones [8].

Discrepancies in the sensitivity to beta-lactam antibiotics were explained by Stock et al. by highlighting the presence of an *ampC* gene similar to those found in other members of the *Enterobacteriaceae* family [8]. Zhou et al. further characterized this antimicrobial resistance gene and designated it as *bla_YOC–1_* encoding for the Class C β-Lactamase YOC-1 [9]. This newly described β-Lactamase displayed an interesting phenotypical profile and was not properly inhibited by clavulanic acid. However, avibactam displayed a strong inhibition against YOC-1 [9]. Zhou et al. also performed an extensive analysis of a multidrug resistance plasmid isolated from a strain of *Y. regensburgei* named pYRW13-125. This plasmid contained 11 resistance genes (*bla_OXA__–10_*, *bla_LAP__–2_*, *dfrA14*, *tetA*, *tetR*, *cmlA5*, *floR*, *sul2*, *ant(3″)-IIa*, *arr-2*, *qnrS1*), conferring multidrug-resistant (MDR) status to this bacterium [9]. Therefore, treating infections caused by these strains may present significant therapeutic challenges in the future. As the antimicrobial resistance phenomenon continues to expand, the medical system might face a new and dangerous threat.

## 3. Structure and Virulence Factors

The information regarding the structure of this Gram-negative bacteria is currently limited. Due to its rare isolation from human clinical samples, it has remained largely overlooked for an extended period. However, with the advancement of modern diagnostic tools, research on *Y. regensburgei* is increasing, driven by the steady rise in its isolation and identification.

In this context, the complex structure of the lipopolysaccharide (LPS) of *Y. regensburgei* is starting to be elucidated. Similar to other Gram-negative bacteria, the LPS serves as the primary virulence factor. The endotoxin is typically responsible for mediating the immune response against this pathogen. The classic structure of the LPS has three different components: lipid A, O-antigen and the oligosaccharide-based core [10]. The O-specific polysaccharide antigen of four strains of *Y. regensburgei* was first described by Jachymek et al. in 1999 through multiple techniques such as high-resolution magic-angle spinning (MAS) nuclear magnetic resonance (NMR) [11]. These findings highlighted a unique structure of the O-antigen in *Yokenlla regensburgei*. Although all examined strains displayed a similar O-antigen structure (basic trisaccharide repeating unit), different immunological properties were revealed. These differences were attributed to the presence of O-acetyl groups substituting certain residues in each structure [11]. Furthermore, Jachymek et al. proved that the approach described in their study could be used in the future for fingerprinting bacteria and other cellular antigens [11].

The oligosaccharide core of the endotoxin is crucial for the biological effect of this virulence factor [10]. The first description of the oligosaccharide core in *Y. regensburgei* was performed by Niedziela et al. [12]. Their work illustrated that this bacterium exhibits a unique core structure with two immunotypes, each recognized specifically by distinct antibodies. The primary difference between these two immunotypes is structural, mainly in the terminal molecules: one features a terminal alpha-D-Glucose while the other one has a terminal alpha-D-Galactose [12]. This structural difference leads to a different spatial arrangement of the molecule and thus different immunogenicity. Usually, the structure of the core in other members of the *Enterobacteriaceae* family involves two regions, an outer core and an inner core. The outer core has a hexose-based structure while the inner core has a more diverse composition, usually with LPS-specific molecules [10,12]. Regarding the electric charge of this structure, *Y. regensburgei* has a negative charge due to the carboxyl residues unlike the other Gram-negative bacteria from the *Enterobacteriaceae* family [12]. The previously highlighted unique structural characteristics may influence the pathogenesis of this bacterium. However, further comprehensive studies are needed in order to draw definitive conclusions.

Another potential virulence factor was described by Kosako et al. in their original paper, confirming the presence of flagella in *Y. regensburgei* through electron microscopy [1]. Flagella typically serve various functions in bacterial biology and virulence, including motility, chemotaxis, adherence to host cells, invasion of host cells, evasion of host immune response and regulating other virulence factors [13]. However, the specific role of flagella in infections produced by this bacterium is currently not known.

Based on the biochemical profile, the presence of several enzymes was presumed. Ying et al. were able to describe a dehydrogenase enzyme from *Y. regensburgei* WZY002 that was able to reduce several aldehydes and ketones used for flavoring in the food industry [14]. Only recently, some genomic evidence regarding the presence of several genes encoding enzymes was unraveled. Meyers et al. managed to describe the presence of genes that encode several enzymes like cellulase, beta-glucosidase, esterase and glutathione S-transferase (GST) in a *Y. regensburgei* isolate [15]. Similarly to flagella, the precise role of these enzymes in the virulence of this bacterium has not yet been extensively studied.

Ever since its discovery, it has been hypothesized that *Y. regensburgei* may possess other virulence factors common to members of the *Enterobacteriaceae* family such as siderophores, capsules, secretion systems, other adherence factors or the ability to produce biofilms [16]. Sahni et al. were able to provide some insights into this issue [17]. In their study, they provided a genomic analysis of *Y. regensburgei* UU2206353 and showed the presence of several genes encoding potential virulence factors like *ompA*, *fur*, *entA*, *entB*, *entS*, *fepA*, *fepC*, *fepG*, *acrA*, *acrB*, *rpoS*, *iroB*, *iroC*, *hcp/tssD*, *rcsB*, *galF*, *gndA*, *ugd*, *iroN* and *bla_YOC-1_* [17]. However, more extensive studies are needed to evaluate their expression and role in the virulence of *Y. regensburgei*.

## 4. An Emerging Pathogen in the Animal World

As previously mentioned, *Y. regensburgei* was initially isolated from the gut of insects from the orders *Hemiptera* (suborder *Heteroptera*—true bugs) and *Coleoptera* (beetles) [1], suggesting an extended animal reservoir from the beginning. Subsequent studies investigating the reservoir of *Y. regensburgei* have increased the understanding of its animal reservoir to include insects such as southern green stink bug (*Nezara viridula*), brown stink bug (*Euschistus heros*), giant spiny stick insect (*Eurycantha calcarata*), termites (*Reticulitermes chinensi*), boxelder bug (*Boisea trivittata*). Addditionally, *Y. regensburgei* has also been isolated from soil samples in China, thus greatly expanding its known natural reservoir [15,18,19,20,21,22,23]. However, veterinary infections produced by this bacterium were not reported until recently. According to the available data, *Y. regensburgei* was isolated from a diverse range of animals including mussels, insects, reptiles and even mammals. However, confirmed infections caused by this bacterium in animals are scarcely reported.

In other invertebrates, *Y. regensburgei* has been previously isolated from various sites. However, its association with specific infections has not been elucidated. Richard et al. investigated mass mortality events in freshwater mussels and were able to highlight some interesting observations [24]. They performed a case–control study on freshwater pheasantshell mussles (*Ortmanniana pectorosa*, previously *Actinonaias pectorosa*—order Unionida) using hemolymph samples and were able to determine that the bacterial loads were higher in moribund mussels compared to healthy ones. Moreover, the bacterial communities described were different, with the moribund mussels displaying fewer sequence variants but a higher relative abundance of proteobacteria including *Y. regensburgei* [24]. Similar results were noted by other articles investigating mussels [24,25,26,27]. These articles described the extent of *Y. regensburgei* in mussel ecosystems, highlighting the diagnostic challenges and its pathogenic potential. However, as stated by the authors, these initial observations require further controlled studies in order to properly assess the involvement of *Y. regensburgei* in mass mortality events in mussels [24]. Some evidence of the pathogenic potential in mussels is addressed by Da Silva Neto et al. in an article evaluating seasonal mass mortality events in freshwater pheasantshell mussles (*Ortmanniana pectorosa*, previously *Actinonaias* pectorosa—order Unionida) [28]. They were able to describe for the first time the foci of hemocytic nodulation and necrosis in these mussels [28].

Similarly, Pawlak et al. were able to identify *Y. regensburgei* from the cloacae of the common grass snakes in Poland (*Natrix natrix*—order Squamata) [29], highlighting another significant reservoir of this bacterium. However, no association between *Y. regensburgei* and a specific infection was described in this case. In 2022, Balamayooran et al. published a paper describing a series of 14 cases of *Y. regensburgei* infections in american alligators (*Alligator mississippiensis*) [30]. In all cases, *Y. regensburgei* was isolated from a wide variety of postmortem samples and was identified using biochemical tests, MALDI-TOF MS and 16S rRNA sequencing. All reptiles from this study succumbed to sepsis, likely originating from the lungs [30]. Based on the different antimicrobial resistance profiles, multiple clones of *Y. regensburgei* were present in the investigated farms [30]. This work highlights the emergence of this new pathogen in reptiles and the importance of correct identification and antimicrobial resistance testing in order to provide proper care in veterinary settings.

An infection produced by *Y. regensburgei* in non-human mammals was recently recorded for the first time by Swaffield et al. in 2023 in a case report of otitis in an australian koala bear (*Phascolarctos cinereus*) caught in the wild [31]. The animal was captured as part of a monitoring project and was initially diagnosed with bacterial cystitis [31]. However, they noticed a purulent discharge from one of the ears and bacteriologic analysis using 16S rRNA sequencing indicated *Y. regensburgei* as the etiologic agent [31]. The infection presented multiple recurrences after the antibiotic treatment was stopped, ultimately requiring surgical intervention. The combined medical and surgical approach of this case led to a favorable outcome and the animal was asymptomatic at six months follow-up. This report provides useful insights into the pathogenic potential of *Y. regensburgei* [31]. Based on the existing data, the emergence of this pathogen has a broader distribution than previously assumed. Veterinary professionals should therefore include this bacterium in their differential diagnosis of bacterial infections.

## 5. Human Infections

Over the years, *Y. regensburgei* was reported as the etiologic agent for several human infections. To properly address the issue of human infections caused by this bacterium, we performed a comprehensive review of the available reported cases. The searches were performed in multiple electronic databases from inception up to the 1st of June 2024: PubMed, Embase, Scopus, Web of Science, Cochrane Library, Med Nar, Google Scholar. Multiple searches were performed in each database using the following simplified wide search strategy and its variations: “(*Yokenella regensburgei* OR *Koserella trabulsii*) AND (human infection)”. Further searches were also performed including terms related to immunosuppression and immunocompetence. The search strategies were initially developed for PubMed and adapted for each individual database. Initial screening was performed by reading the title and abstract of each study. Full-text articles were evaluated by two researchers D.A.T. and V.S.N. Discrepancies were resolved through discussion and by expert oversight from two authors, C.C. and I.A.C. All case reports or series of case reports, available online, regarding human *Y. regensburgei* infections written in English, Spanish and French were included in the qualitative review. We excluded other types of articles (such as book chapters, review articles, etc.) and articles written in languages other than English, Spanish, or French, or those not focusing on human infections caused by *Y. regensburgei*. References of included articles were screened and subsequent relevant studies were included in the review. Automated tools were not used during the search, screening or data extraction steps of the qualitative review.

A total of 22 articles were included in the qualitative review, containing 23 reported cases of human infections (Table 1). With one exception, all included studies were case reports and only one was a case series containing two cases. The analysis was structured according to the infection site in the following sub-chapters: skin and soft tissue infections, bacteriemia and sepsis, osteoarticular infections and other infections.

The antimicrobial resistance patterns of the isolated strains of *Y. regensburgei* from the included studies are outlined separately in Table 2. A total of 15 cases described the antibiotic resistance profile of *Y. regensburgei*, with most data highlighting resistance to multiple β-Lactam antibiotics (penicilins and cephalosporins, including combinations with beta-lactamase inhibitors: amoxicillin–clavulanate) and in a smaller proportion to others such as tetracycline, trimethoprim-sulfamethaxazole, nitrofurantoin and colistin. In all instances where information was available, the guidelines provided by the CLSI (Clinical and Laboratory Standards Institute) were utilized for interpreting the antimicrobial susceptibility profile. Overall, most included studies lacked any information regarding the susceptibility guidelines used for the interpretation of the antibiotic resistance profile. Consequently, no definitive conclusions can be drawn regarding the antibiotic resistance profile of *Y. regensburgei* based on the included cases. Further studies are required in order to systematically address this issue in the future.

### 5.1. Skin and Soft Tissue Infections

*Y. regensburgei* has been isolated from various infection sites, most frequently from skin or soft tissue samples. The typical presentation consisted of a wound accompanied by non-specific local and systemic signs of infection: fever, pain, edema and local heat. Most described lesions described were located on the lower limbs, some presumably resulting from contact with soil during farming or gardening activities. However, due to the significant variability in the descriptions of skin and soft tissue infections caused by *Y. regensburgei*, a common clinical presentation was not evident from the reviewed cases.

*Y. regensburgei* was identified in fluid drawn from vesicles surrounding a perimalleolar ulcer by Fajardo Olivares et al. [32]. Cellulitis determined by this pathogen was diagnosed in two other cases in 2011 and 2013 by Lo et al. and Bhowmick et al. respectively [35,37]. In the second case, cellulitis was associated with lymphangitic streaking, reminiscent of *Streptococcus pyogenes* infections [37]. It was also the only case in which the presence of yellowish exudate was noted, prior to hospital presentation. The authors described the lesion as a bulla with ”a scarlatiniform lacy rash”. In 2019, Wright et al. noted a completely different presentation—multiple areas of purpuric lesions, with grayish necrotic fascia and extensive subcutaneous tissue necrosis upon surgical debridement [44]. One of the more recent case reports described by Huang et al. in 2022 contains no description of the appearance of the lesion [49]. AlMutawa et al. reported a chronic draining wound infection in an immunocompetent individual associated with elbow trauma after a mountain bike accident. In this case, the wound was localized, with limited erythema, pain and no signs of osteomyelitis. The occurrence of post-traumatic wound infection further suggests that contact with soil might be a significant risk factor for skin and soft tissue infections caused by *Y. regensburgei* [51].

Regarding the treatment, the first antibiotic used in 1994 for a *Y. regensburgei* infection was amikacin, with no further information available regarding the outcome of the patient [7]. Intravenous clindamycin was part of the therapeutic scheme in two of the more severe reported cases [37,44]. Clindamycin was paired with intravenous vancomycin and cefepime in one case, failing to improve the evolution of necrotizing fasciitis, ultimately leading to amputation. In the other case, clindamycin was paired with imipenem-cilastatin and gentamicin, but the patient died on the night of admission. Third-generation cephalosporins were part of successful treatments, intravenous ceftriaxone alone in the case reported by Lo et al. and accompanied by sulbactam and levofloxacin in the more recent case [35,49]. Another fluoroquinolone, namely ciprofloxacin was the therapeutic strategy chosen in 2005, resulting in the disappearance of vesicles and no signs of pain, though the primary lesion persisted [32]. One of the more recent cases reported by AlMutawa et al. required multiple antibiotic treatment courses (3 days IV ceftriaxone, three courses lasting 7–10 days each of oral amoxicillin/clavulanate combined with cefalexin) that did not resolve the infection [51]. The infection was eventually resolved by a combination of local surgery and drainage (with the removal of a wood fragment) followed by an antibiotic regimen of sulfamethoxazole/trimethoprim lasting two weeks. This case further highlights the possible contamination with *Y. regensburgei* from the environment through wounds and the need for correct bacterial identification [51].

### 5.2. Bacteremia and Sepsis

Bacteriemia can be defined as the presence of viable bacteria in the bloodstream [52]. Transient bacteriemia is fairly common during normal activities and in most cases these findings are benign and do not lead to complications [52]. The evolution towards sepsis occurs only when the immune mechanisms are overwhelmed. Sepsis is a life-threatening condition characterized by multiple organ dysfunction resulting from physiological and pathological abnormalities. The subsequent aberrant immune response of the host is influenced by both pathogen- and host-related factors and can lead to septic shock, further increasing mortality [52,53,54].

In most of the cases previously mentioned, isolation of *Y. regensburgei* was performed from blood samples and bacteremia accompanied the main suite of manifestations described. The approach towards sample collection varied in terms of the number of samples per patient and the timespan between them—one blood specimen [7], two at 2 h intervals [35] or four at 1 h intervals [41]. Regardless of the collection method, at least two blood cultures were mentioned to be positive as part of the diagnosis scheme. The time between inoculation and obtaining a positive culture was also variable. Moreover, bacterial growth was not consistently obtained within a standard time frame. In one case, blood cultures were still negative after 120 h of incubation at 37 °C [44]. In another case, the absence of bacterial growth after 48 h of incubation prompted the analysis of a second sample, which became positive within 48 h. The first sample subsequently showed growth after five days [36]. Another case was reported by Fukatsu et al. detailing bacteriemia in a 74-year-old diabetic patient with foot gangrene that required amputation of both lower limbs. Wound swabs and blood cultures performed after the amputation revealed bacterial growth identified through MicroScan biochemical testing, MALDI-TOF MS and 16s rRNA sequencing as *Y. regensburgei* [43]. Multiple antibiotic regimens were administered in this case including clindamycin, meropenem, piperacillin-tazobactam, doripenem (as post-surgical prophylaxis) and ampicilin-sulbactam combined with ceftazidime for nine days following bacterial identification and susceptibility testing. The patient improved and *Y. regensburgei* could not be further evidenced following one week of ampicilin-sulbactam and ceftazidime [43].

In the paper published in 1994, Abbott et al. described a second case of infection with *Y. regensburgei*, particularly because it was only associated with transient bacteremia [7]. In the reported case, a female patient was admitted to the hospital for gastrointestinal bleeding. Alongside other investigations, a blood sample was drawn. Isolation of a Gram-negative rod prompted treatment with ciprofloxacin, the patient was released, with no follow-up information available. Later laboratory analysis identified the bacteria as *Y. regensburgei* [7].

Although bacteremia was observed in several cases, the occurrence of sepsis was less common. Chi et al. 2017 reported sepsis as the main manifestation of a *Y. regensburgei* infection [41]. The patient in question presented to the hospital with a fever and absence of chills. Physical examination revealed hypertension, tachycardia, and tachypnea, and correlated blood work raised the suspicion of severe sepsis. These findings prompted treatment with cefoxitin and dexamethasone, coupled with blood transfusions and erythropoietin administration. The patient was eventually discharged, and no recurrence was reported at one-year follow-up [41]. Two other cases presented patients with sepsis associated with a skin or soft tissue infection—cellulitis and purpuric lesions, later proved to be necrotizing fasciitis [37,44].

### 5.3. Osteoarticular Infections

*Y. regensburgei* has been identified in several cases involving bone and joint infections. The earliest documented case of a musculoskeletal infection determined by *Y. regensburgei* infection was reported in 1994 from a septic knee [7]. Another case was described by Penagos et al. in 2015 [38]. The patient presented with signs of osteomyelitis six weeks following surgical intervention of an invasive pituitary macroadenoma. Ipsilateral eyelid ptosis and periorbital inflammation were observed. A postoperative infectious complication was presumed and, in this case, osteomyelitis was treated with cefazolin and clindamycin [38]. However, after the isolation of the bacteria from fluid collections in the epidural and subdural areas, the treatment regimen was changed to intravenous ciprofloxacin. Following treatment, the patient was discharged, with no recurrence of infection at one-year follow-up [38]. Another case was reported by Lee et al. in 2015 highlighting a diabetic foot infection in a Korean patient [40]. The patient presented with a history of diabetes, chronic kidney disease (previous kidney transplant and hemodialysis) and recent osteomyelitis. The patient was admitted with toe gangrene, which was treated with angioplasty, debridement, disarticulation and IV cefotetan [40]. Exudate and tissue specimens were cultured in multiple instances and the resulting bacterial growth was further identified through VITEK 2 revealing a possible polymicrobial infection: *Pseudomonas aeruginosa*, *Citrobacter freundii*, and *Enterobacter cloacae* (first inoculation), *P. aeruginosa* and *C. freundii* (post-surgery day four), *P. aeruginosa* and *Y. regensburgei* (post-surgery day seven), *P. aeruginosa* (post-surgery day twenty). The identification of *Y. regensburgei* was further confirmed through 16S rRNA and *gyr*B sequencing [40]. The patient was treated with piperacillin-tazobactam for at least 20 days, but the progression of the necrotic lesions prompted surgical amputation of the leg in order to resolve the infection. As suggested by the authors, in this particular case, the pathogenic potential of *Y. regensburgei* could not be definitively proven, given the simultaneous identification of other well-known pathogens [40].

A more recent case involving osteomyelitis was published in 2021 [46]. The patient, suffering from both diabetes and rheumatoid arthritis, had undergone three surgical procedures over the course of less than three years: primary total arthroplasty of the knee with a bone graft, which required replacement of the mobile parts of the implant two months later and complete removal of the prosthetic two years and seven months after [46]. *Y. regensburgei* was isolated from both a bone fragment during the second surgery and from purulent secretions collected during the third procedure. The first treatment regimen consisted of meropenem for two weeks and oral sulfamethoxazole/trimethoprim in the four weeks following improvement [46]. The second treatment regimen included intravenous sulfamethoxazole/trimethoprim for seven days with significant improvements, followed by an oral regimen of another six months, due to suspicion of chronic osteomyelitis [46].

Finally, osteomyelitis was diagnosed in a case described by Denes et al. in 2021 [47]. The infection concerned the last phalanx of the second finger and required surgical debridement, during which bacteriological samples were collected. Their later examination revealed *Y. regensburgei* to be the etiologic agent and the patient was started on ofloxacin and co-trimoxazole for three months. Improvement was noted on a two-month follow-up and no relapse was reported two months after the completion of the treatment [47].

### 5.4. Other Infections

Even though most cases of infection with *Y. regensburgei* involved the presence of wounds or skin lesions, there are a few exceptions in which the mentioned pathogen was incriminated.

Rinonos et al. described a case of an intracranial abscess associated with *Y. regensburgei* in a patient diagnosed with primary Central Nervous System (CNS) lymphoma [45]. One month after craniotomy and partial resection, the patient underwent the first cycle of chemotherapy, shortly thereafter developing incisional purulence, followed by progressive lethargy. *Y. regensburgei* was identified using MALDI-TOF MS and the VITEK 2 Automated System. The immunosuppression caused by chemotherapy, along with exposure to well water and poor hygiene, likely increased the risk of contamination with *Y. regensburgei* in this case [45].

To the extent of our knowledge, the first reported case in which *Y. regensburgei* was isolated from the external auditory canal was described by Na et al. in 2021 [48]. The chief complaint of the patient was odorless otorrhea and mild pain in his right ear. After more investigations, swelling and debris accumulation were identified, but the tympanic membrane was intact with no hearing loss, dizziness or fever present [48]. In order to treat the infection, ciprofloxacin was prescribed, and the patient made a full recovery [48]. It is worth mentioning that these symptoms appeared after the patient went scuba diving in Gapyeong Valley, South Korea, which may be the site where contact with the pathogen occurred [48].

*Y. regensburgei* was isolated from urinary tract infections (UTIs) in multiple instances. Aziz et al. identified *Y. regensburgei* through VITEK 2 in a urine sample from a patient with chronic kidney disease without providing other details regarding the case [39]. Another particular case of infection produced by *Y. regensburgei* was described by Sheeba et al. in 2023, which manifested as a urinary tract infection [50]. The patient complained of common UTI symptoms (increased frequency of urination, burning sensation during micturition and dribbling, with the absence of fever) and had a history of multiple UTIs, benign prostatic hypertrophy and urinary symptoms such as incomplete voiding, poor urine flow and urinary retention [50]. He had undergone a transurethral prostate resection 5 years prior and a urethral dilatation 2 years prior. The treatment scheme consisted in a combination of cefoperazone–sulbactam and scheduling the patient for a substitution urethroplasty [50]. The most recent uncomplicated UTI case was reported by Sahni et al. The authors identified *Y. regensburgei* both from the urine and suprapubic aspirate of a 69-year-old patient with previous benign prostatic hypertrophy through MALDI-TOF MS [17].

Similarly, Semler et al. previously described a case of UTI complicated with sepsis in a patient previously diagnosed with type I diabetes mellitus complicated by renal failure [33]. The patient had undergone two heterotopic renal transplants, the most recent being five years prior. The patient presented to the emergency department with a three-day history of back pain, nausea, vomiting, low-grade fevers, and night sweats. Later, the patient was admitted to the general medicine service, where urine and blood samples were collected for bacterial cultures. A complicated urinary tract infection was presumed due to the immunosuppressive treatment and antibiotic treatment with piperacillin/tazobactam was initiated. On the second day of hospitalization, the patient became febrile (39.8 °C) with a white blood cell (WBC) count of 12,000 cells/mm^3^ [33]. Given his renal history and current condition, a renal ultrasound was performed, showing a normal renal aspect with no signs of hydronephrosis or perinephric fluid, combined with a declining renal function. Ultimately, the patient met the criteria for sepsis, and his urinary electrolytes indicated a prerenal cause [33]. Blood and urine cultures were positive for Gram-negative rods, and *Y. regensburgei* was identified using the VITEK 2 system [33]. Although the treatment with piperacillin/tazobactam initially improved the patient’s condition, the antimicrobial susceptibility pattern prompted a modification of the treatment regimen, ultimately administering oral ciprofloxacin 250 mg every 12 h for 14 days [33]. The patient was discharged with no recurrence of infection during a three-month follow-up period [33].

Another anatomical site from which *Y. regensburgei* was isolated was the gastrointestinal tract. In 2017, Milori A. reported a case of diarrhea caused by *Y. regensburgei* [42]. At the time of presentation, the patient presented with a three-day history of 6–7 episodes of diarrhea daily, accompanied by fever and chills. Stool samples were analyzed and revealed a high count of white blood cells, erythrocytes and subsequent stool cultures revealed *Y. regensburgei* as the cause of this infection. Treatment consisted of cefixime, resulting in discharge of the patient three days later and no relapse on follow-up, after six more days of medication. In addition, another case of infection with *Y. regensburgei*, involving the gastrointestinal tract was presented by Fill et al. in 2009 [34]. The patient underwent esophagogastrostomy for esophageal adenocarcinoma. Postoperatively, the patient developed a liver abscess and became septic [34]. The treatment regimen consisted of a combination of several antibiotics and antifungals such as levofloxacin, linezolid, fluconazole, and metronidazole [34]. Given the absence of evidence for an anastomotic leak, it is believed that immunosuppression played a role in this case [34].

## 6. Future Perspectives

With the new research tools becoming increasingly available, we witness an overwhelming new set of data unraveling regarding this pathogen. These exciting new findings might shed new light on the biology, metabolism and involvement of *Y. regensburgei* in human and veterinary infections.

*Y. regensburgei* could become invaluable in future efforts to combat different types of pollution such as plastic pollution and heavy metal contamination of wastewater. A Korean team of researchers led by Park et al. made an extraordinary discovery regarding the biology of *Y. regensburgei* [23]. They successfully isolated this bacterium from mealworm larvae (*Tenebrio molitor*) and demonstrated its ability to degrade polystyrene [23]. Their work marks a premier regarding the biology of this bacterium since there are no other reports that describe this phenomenon [23]. Additionally, the *Y. regensburgei* strain GXAS49-I, isolated from soil samples, has demonstrated the ability to effectively remove heavy metals such as Cu and Pb through absorption and biomineralization processes. This suggests its potential use in removing heavy metals from wastewater [22].

Another notable finding was presented by Palmisano et al., who evaluated changes in gut microbiota composition following various bariatric surgeries. [55]. Their findings demonstrated that the abundance of *Y. regensburgei* increased and remained stable throughout the study in patients who underwent Roux-en-Y gastric bypass [55]. In light of the accumulating evidence supporting the pathogenic potential of *Y. regensburgei*, it is crucial for medical professionals to be well-informed about this bacterium to effectively address any resulting infections it may cause.

More recently, Liu et al. performed a genome-wide association study in order to analyze the relationship between the nasal microbiome and various human genetic variations [56]. Using data from a cohort of 1593 patients from China, they identified a noteworthy phenotypic correlation [56]. They proved a causal relationship between *Y. regensburgei* and certain cardiometabolic markers, specifically glutamic acid and creatine concentrations [56]. Their study could have significant future implications, highlighting the link between the microbiome and different metabolites with roles in cardiometabolic health. This research could lead to new treatment strategies targeting the microbiome, potentially improving patient outcomes.

## 7. Conclusions

*Yokenella regensburgei* can be considered a rare and emerging pathogen in human and veterinary medicine. With a robust natural reservoir and the increased availability of new and improved diagnostic tools worldwide, a future rise in the number of clinical isolates can be expected. Currently, different automated diagnostic tools such as MALDI-TOF MS and VITEK 2 can accurately identify *Y. regensburgei*. Due to the insufficiently known antimicrobial resistance profile and understudied virulence factors, the medical community must be prepared to properly address this issue. At the moment of writing, most reported human cases seem to be associated with immunosuppression (alcohol abuse, diabetes, chronic kidney disease, etc.). However, some cases have also been reported in patients with no known immune suppression, underlying the unknown virulence potential of this bacterium. These facts highlight the need for further research into the pathogenic potential and antibiotic susceptibility of *Y. regensburgei*.

## Figures and Tables

**Table 1:** Human *Yokenella regensburgei* infections.

No.	Reference	Country	Year of Publication	Patient—Age (y.o.)/Sex	Sample	Type of Infection	Bacterial Identification	Antibiotic Treatment	Outcome
1	Abbott et al. [7]	USA	1994	74/M	Wound	Septic knee	VITEK 2	Amikacin	N/A
				35/F	Blood	Transient bacteremia		Ciprofloxacin	N/A
2	Fajardo Olivares et al. [32]	Spain	2005	82/M	Vesicle fluid	Perimalleolar ulcer	Biochemical tests and MicroScan Walkaway	Ciprofloxacin	S
3	Semler et al. [33]	SUA	2008	57/M	Blood, urine	Urinary tract infection complicated with sepsis	VITEK 2	Ciprofloxacin	S
4	Fil et al. [34]	SUA	2009	77/M	Abdominal fluid, blood and sputum	Liver abscess	MicroScan Walkaway	Piperacillin-tazobactam, levofloxacin, linezolid, metronidazole	S
5	Lo et al. [35]	Taiwan	2011	42/M	Blood	Wound infection	VITEK 2, BP Phoenix, Voges–Proskauer reaction	Amoxicillin-clavulanate, ceftriaxone	S
6	Jain et al. [36]	India	2013	5/M	Blood	Enteric fever	VITEK 2	Ciprofloxacin	S
7	Bhowmick et al. [37]	USA	2013	48/M	Bulla aspirate, Blood	Celulitis complicated with sepsis	MicroScan Walkaway, ribosomal sequence, BD Phoenix	Imipenem, clindamycin, gentamicin	D
8	Penagos et al. [38]	Colombia	2015	70/F	Fluid colections	Osteomyelitis	VITEK 2 and BD Phoenix	Cefazolin, clindamycin, ciprofloxacin	S
9	Aziz et al. [39]	Iraq	2015	N/A	Urine	Chronic kidney infection	VITEK 2	N/A	N/A
10	Lee et al. [40]	Korea	2015	71/M	Tissue, wound exudate	Diabetic foot infection	Vitek 2, 16s rRNA and *gyr*B sequencing	Cefotetan, piperacillin-tazobactam	S
11	Chi et al. [41]	China	2017	38/M	Blood	Sepsis	MicroScan Walkaway	Cefoxitin	S
12	Milori et al. [42]	Greece	2017	17/M	Stool	Diarrhea	VITEK 2	Cefixime	S
13	Fukatsu et al. [43]	Japan	2017	74/M	Blood, wound	Bacteriemia, diabetic foot gangrene	Biochemical tests, MALDI-TOF MS, 16s rRNA	Ampicillin-sulbactam, Ceftazidime	S
14	Wright et al. [44]	Japan	2019	64/F	Blood, Wound	Necrotizing fasciitis	BD Phoenix	Vancomycin, cefepime, clindamycin	S
15	Rinonos et al. [45]	SUA	2020	67/F	Intraoperative brain abscess specimen	Bran abscess	MALDI-TOF MS and VITEK 2	Carbapenems and fluoroquinolones	S
16	Guilarde et al. [46]	Brazil	2021	54/F	Pus	Septic knee	VITEK 2	Meropenem, sulfamethoxazole/trimethoprim	S
17	Denes et al. [47]	France	2021	71/M	Swab	Osteomyelitis	N/A	Ofloxacin, sulfamethoxazole/trimethoprim	S
18	Na et al. [48]	Korea	2021	56/M	Swab from otorrhea	Otitis	MALDI-TOF MS, 16S rRNA, VITEK 2	Ciprofloxacin	S
19	Huang et al. [49]	China	2022	72/F	Pus	Wound infection	MALDI- TOF MS	Cefoperazone–sulbactam, levofloxacin	S
20	Sheeba et al. [50]	India	2023	69/M	Urine	Urinary tract infection	Biochemical tests and MALDI-TOF MS	Cefaperazone-sulbactam	S
21	AlMutawa et al. [51]	Canada	2023	38/M	Wound secretions	Wound infection	MALDI-TOF MS	Sulfamethoxazole + trimethoprim	S
22	Sahni et al. [17]	India	2024	69/M	Urine, suprapubic aspirate	Urinary tract infection	MALDI-TOF MS, whole genome sequence	N/A	N/A

No., Case number; N/A, Not available or no follow-up; M, Male; F, Female; S, Survived; D, Died.

**Table 2:** Antimicrobial resistance profile of *Y. regensburgei* isolates from human infections.

No.	Reference	AST Guideline	Antimicrobial Resistance Profile
1	Abbott et al. [7]	N/A	N/A
2	Fajardo Olivares et al. [32]	N/A	N/A
3	Semler et al. [33]	N/A	I: ampicilin
4	Fil et al. [34]	N/A	N/A
5	Lo et al. [35]	CLSI	R: ampicilin, amoxicillin–clavulanate and cefazolin
6	Jain et al. [36]	CLSI	R: penicillin, cefoxitin and colistin
7	Bhowmick et al. [37]	N/A	N/A
8	Penagos et al. [38]	N/A	N/A
9	Aziz et al. [39]	N/A	R: nitrofurantoin, tetracycline and trimethoprim-sulfamethaxazole
10	Lee et al. [40]	CLSI	R: ampicilin, amoxicillin–clavulanate, cefoxitin, cefotaxime and cefuroxime
11	Chi et al. [41]	CLSI	R: ampicilin and piperacilin I: amoxicillin–clavulanate
12	Milori et al. [42]	CLSI	R: ampicilin and colistin I: amoxicillin–clavulanate
13	Fukatsu et al. [43]	N/A	R: amoxicillin–clavulanate, cefaclor, cefazolin, cefotiam and flomoxef I: ampiclin, cefmetazol
14	Wright et al. [44]	N/A	R: ampicillin and cefazolin
15	Rinonos et al. [45]	N/A	R: ampicillin and cefazolin
16	Guilarde et al. [46]	CLSI	R: cefazolin
17	Denes et al. [47]	N/A	N/A
18	Na et al. [48]	N/A	R: ampicillin, cefazolin, and amoxicillin–clavulanate
19	Huang et al. [49]	CLSI	N/A
20	Sheeba et al. [50]	CLSI	R: penicillin, amoxicillin, ampicillin, amoxicillin-clavulanate, cefoxitin and colistin
21	AlMutawa et al. [51]	CLSI	R: cefazolin and ceftazidime I: ampicilin
22	Sahni et al. [17]	CLSI	R: penicillin, aminopenicillins, cefoxitin, and colistin

Abbreviations: AST, Antimicrobial Susceptibility Testing; CLSI, Clinical and Laboratory Standards Institute; N/A, not available; R, resistant; I, intermediate.

## Data Availability

All data is contained within the article.

## Abbreviations

D.A.T., Dan Alexandru Toc; C.C., Carmen Costache; V.S.N., Vlad Sever Neculicioiu; I.-M.R., Irina-Maria Rusu; B.-V.R., Bogdan-Valentin Roznovan; A.B., Alexandru Botan; A.G.T., Adelina Georgiana Toc; P.S., Pavel Șchiopu; P.-S.P., Paul-Stefan Panaitescu; A.G.P., Adrian Gabriel Pană; I.A.C., Ioana Alina Colosi.
